# Blade Rub-Impact Fault Identification Using Autoencoder-Based Nonlinear Function Approximation and a Deep Neural Network

**DOI:** 10.3390/s20216265

**Published:** 2020-11-03

**Authors:** Alexander E. Prosvirin, Farzin Piltan, Jong-Myon Kim

**Affiliations:** Department of Electrical, Electronics and Computer Engineering, University of Ulsan, Ulsan 44610, Korea; alpros91@mail.ulsan.ac.kr (A.E.P.); pantea1384@mail.ulsan.ac.kr (F.P.)

**Keywords:** data-driven fault diagnosis, deep autoencoder, deep neural network, deep learning-based function approximation, fault diagnosis, rotating machinery, rub-impact fault, nonlinear-based fault diagnosis

## Abstract

A blade rub-impact fault is one of the complex and frequently appearing faults in turbines. Due to their nonlinear and nonstationary nature, complex signal analysis techniques, which are expensive in terms of computation time, are required to extract valuable fault information from the vibration signals collected from rotor systems. In this work, a novel method for diagnosing the blade rub-impact faults of different severity levels is proposed. Specifically, the deep undercomplete denoising autoencoder is first used for estimating the nonlinear function of the system under normal operating conditions. Next, the residual signals obtained as the difference between the original signals and their estimates by the autoencoder are computed. Finally, these residual signals are used as inputs to a deep neural network to determine the current state of the rotor system. The experimental results demonstrate that the amplitudes of the residual signals reflect the changes in states of the rotor system and the fault severity levels. Furthermore, these residual signals in combination with the deep neural network demonstrated promising fault identification results when applied to a complex nonlinear fault, such as a blade-rubbing fault. To test the effectiveness of the proposed nonlinear-based fault diagnosis algorithm, this technique is compared with the autoregressive with external input Laguerre proportional-integral observer that is a linear-based fault diagnosis observation technique.

## 1. Introduction

A blade rub-impact fault is a severe type of mechanical fault frequently occurring in rotating machinery, especially in various turbines. The interactions between the blades of the rotor and the stationary parts of rotating machines can be recognized as a separate mechanical fault that can be caused by rotor blade extension due to the high operating temperatures or as a coupling fault where the rub-impact is a consequence (or evidence) of a different mechanical fault. Under the faults leading to blade rub, usually, shaft imbalance, misalignments, excessive self-excited vibrations, or bearing failures are understood [1]. If not detected and identified at the early stages, a blade rub-impact fault may cause the failure of the system and severe economic loss.

Vibration signal analysis [2] is most frequently applied for diagnosing blade rub-impact faults in comparison with other methods, such as acoustic [3], pressure [4], and temperature analysis [5]. The main reason for its application is that performing the vibration signal acquisition in the field is relatively easy compared to other techniques. However, it is known that for proper vibration analysis, the signal processing methods play an essential role. A system with a rotor-to-stator rub impact fault is recognized as a complex nonlinear and nonstationary one [6,7]. This is because, simultaneously, several physical processes are involved in the process of rubbing, such as vibration, thermal effects, stiffness, and friction [8]. These facts cause limitations in the application of the conventional time- and frequency-domain analysis approaches based on statistical feature extraction and Fourier transforms, and they force researchers and engineers to utilize complex time-frequency analysis (TFA) methods for extracting relevant information about the mechanical fault and performing fault diagnosis [9].

In recent years, many studies focused on the extraction of discriminative features from rotor systems using TFA approaches for diagnosing blade rub-impact faults. The most frequently used TFAs are empirical mode decomposition (EMD) [10] and its derivative methods, such as ensemble EMD (EEMD) [11], Hilbert–Huang Transform (HHT) [12], and wavelet transform with its variations [13,14], including Harmonic Wavelet Transform [15]. All these methods appeared to be capable of effectively extracting valuable fault features from nonlinear and nonstationary rotor systems in general, and systems with blade rub-impact faults, specifically. However, these methods suffer some drawbacks that lead to difficulties in their application in real industrial fields. First, the EMD method suffers from the problem of mode-mixing [16], which means that multiple oscillating components are presented in a single intrinsic mode or similar oscillating components are getting split into several modes with disrupted amplitudes. This problem causes difficulties in the interpretation of the decomposition results and affects fault diagnosis accuracy. Second, although EEMD successfully resolves the problem of mode mixing, it drastically increases the computational complexity of this iterative algorithm. It runs an ensemble of EMD decompositions on a signal to achieve “clear” intrinsic modes. This leads to problems in using this approach in industrial applications, especially when near real-time performance is needed. Finally, talking about the wavelet transform family of methods, they suffer from energy leakage and interference terms that make it difficult to interpret the results of decomposition. Furthermore, the selection of the mother wavelet function significantly affects the results of the wavelet transformation [17]. Unfortunately, to find an appropriate mother wavelet function that correlates well with the signal properties, a series of experiments is needed that leads to subjectivity in this selection process and makes it difficult to make this type of analysis adaptive [18].

For decision making, machine learning algorithms have been extensively utilized in the fields of fault diagnosis and condition monitoring. As examples of the classical widely applied machine learning methods in this domain, k-nearest neighbors [19], support vector machines (SVM) [20], decision trees [21], and shallow artificial neural network (ANN) [22,23] architectures can be considered. These algorithms use different concepts for learning how to perform the task of classification, but they have one thing in common: the machine learning algorithms in the field of fault diagnosis are mainly trained on the manually chosen or hand-crafted features to diagnose the mechanical faults. In this case, the fault diagnosis performance of the classical machine learning algorithms relies strongly on human knowledge and expertise, which cannot guarantee that these features are optimal and best characterize the specific fault type being investigated.

Due to the complexity of implementation and the problems of TFA signal analysis approaches for extracting discriminative fault features as well as the problems of the classical machine learning algorithms that are dependent on the feature quality, the other family of algorithms is widely used in the industry for assessing the health condition of rotor systems. These algorithms belong to the family of control theory-based algorithms. Thus, linear-based observers such as proportional–integral observer (PIO) and proportional multi-integral observer (PMIO) have been successfully used in several applications [24,25]. Despite the various advantages of the linear-based observer such as the simplicity of implementation in industrial applications and their flexibility, the main challenges of this method are robustness and reliability. To address the issues of linear-based observers, two different schemes have been defined by researchers: designing the nonlinear-based observer and artificial intelligence-based observation techniques. The nonlinear-based observation techniques such as sliding mode fault observer, feedback linearization fault observer, and backstepping fault observer are used in several applications. Apart from the various advantages of nonlinear-based observers such as stability, reliability, and robustness, these techniques suffer from its complexity [26,27]. The second scenario is based on improving the linear-based observer performance using artificial intelligence-based techniques. This scenario also can be implemented in two ways. The first way is to improve the fault diagnosis performance of the conventional linear-based observation techniques with the addition of artificial intelligence techniques into the different stages of the fault diagnosis pipeline [21,28]. In another way, the nonlinear rotor systems can be diagnosed in a data-driven manner by replacing linear-based observation techniques with artificial intelligence-based approaches, such as using methods from the field of deep learning that are easier to implement and set up compared to the modern nonlinear observation techniques from control theory. This is the scenario that has been chosen in this work.

Recently, the deep learning-based approaches for fault feature extraction and fault classification are drawing attention due to the increased computational power. The deep learning field provides us with a variety of techniques that are capable of learning the discriminative features autonomously or generating them from the given data representations and are actively applied for condition monitoring and predictive maintenance. For instance, talking about supervised learning, convolutional neural networks [29] autonomously extract high-level features from the images [30,31] and one-dimensional signals [32,33]. Another technique from the supervised learning family is called deep neural network (DNN) [34], which resembles the conventional ANN with a difference in the depth of the network and number of neurons in its layers, and it also offers better classification capabilities when dealing with high-dimensional input data (can be a one-dimensional signal instead of feature parameters) and uncertainties within it compared to the conventional machine learning algorithms, including shallow structured ANNs. Furthermore, to fully understand the capabilities of deep learning, it is important to discuss the unsupervised methods that are introduced by researchers. One of the great examples of unsupervised deep learning techniques is generative adversarial networks (GANs) [35,36]. GANs learn the statistical parameters of the input data distribution and are capable of generating new data of similar distribution. In the field of fault diagnosis, GANs are frequently utilized for solving the problem of fault data augmentation before training DNNs for decision making [37]. This application is reasonable and draws a lot of attention in the industry, since it is usually not possible or even expensive to collect huge datasets of faulty signals from real applications. However, some challenges might cause difficulties when applying GANs to mimic nonstationary signals of the nonlinear system. First, as was mentioned above, GANs learn the distribution of the input signals during the training so they can generate sequences of similar distribution from the random noise. It is known that in nonstationary signals, the statistical parameters of the time sequences tend to change with the time even when these signals are collected during the same operating condition. Hence, it can be a difficult problem to learn the exact properties of the nonlinear system using GANs. Second, some external disturbances from the environment affect the recorded signals, and this unwanted noise will be also included in the distribution learned by GAN. Finally, it is understandable that the learned distribution also depends on the length of time sequence. To address or avoid these problems, the indirect approximation (i.e., the approximation of the system state by learning the specific features of the system instead of signal distribution) of a nonlinear system can be useful. Autoencoders (AEs) [38] are another type of deep learning technique that can be used to resolve this issue. During the training, AEs learn the specific nonlinear discriminative features (latent coding) of the input data that allow us to use them for feature extraction to determine the state of the system, data reconstruction, data generation, compression, and fault detection in the system [39,40]. Furthermore, there are extensions of the conventional AEs called denoising autoencoders (DAEs) that enhance the capabilities of AEs to learn the discriminative features of the system by the signals even when those are highly contaminated by noise [41].

Considering the information gathered from the literature review, in this paper, we propose a novel method based on a deep undercomplete denoising autoencoder (DUDAE) and a DNN to address the issues of approximating the nonlinear function of the rotor system with coupling blade rub-impact faults and to perform fault identification in a data-driven manner. In this work, we attempt to replace the conventional two-block-based control theory methodology for estimating signal behavior (function approximation of the system and signal estimation) with one block, which is represented by the deep learning technique DUDAE. First, the DUDAE is trained using the vibration signals corresponding to the healthy state of the rotor system. During this step, the DUDAE learns the latent coding in its bottleneck layer that represents the nonlinear function of the rotor system under normal operating conditions. Next, the vibration signal corresponding to the unknown state of the rotor system is pushed at the input layer of the DUDAE, where it estimates the signal of the current state using the latent coding learned on signals of normal operating conditions. Then, the residual signal (i.e., error signal) is generated as the difference between the real vibration signal of an unknown system state and the one estimated by the DUDAE. Residue generation is used for enhancing the dissimilarities of the signals corresponding to different classes using the anomaly detection properties of the autoencoder, and hence, it generates sequences (residual signals) that are treated as discriminative features capable of improving fault diagnosis performance. At the final step, these residual signals are used as inputs to the DNN to accomplish the task of fault identification of rotating machinery.

The specific contributions of this study can be summarized as below:

1. The novel data-driven method for diagnosing coupling rotor imbalance and blade rub-impact faults in nonlinear rotor systems is presented.
2. The deep learning-based system identification approach for approximating the nonlinear function of the system and state estimation has been introduced as a part of the proposed fault diagnosis solution.

The remainder of this manuscript is organized as follows. Section 2 introduces the proposed methodology for diagnosing the coupling blade rub-impact faults of different severity levels. Section 3 provides an experimental validation of the introduced framework and discussion. Finally, Section 4 contains the concluding remarks.

## 2. Proposed Methodology

**Figure 1:**
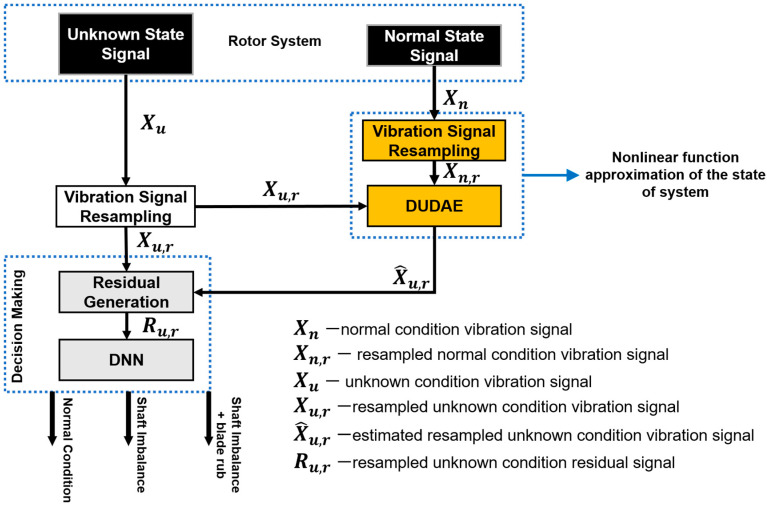
The proposed framework for the assessment of the health state of the rotor system.

A block diagram of the proposed approach for identifying the coupling blade rub-impact faults of various intensities is depicted in Figure 1, and it consists of three important steps. First, the collected vibration acceleration signals corresponding to the normal operating state when no faults are observed in the system are used to train the DUDAE to create a nonlinear function approximation of a system under normal operating conditions. Then, the autoencoder’s property of anomaly detection is used to represent the deviations in the state of the system by generating the residual signal. This residual signal represents the difference (error) between the current vibration signal approximated by the DUDAE using the learned nonlinear function of the normal state of the system and the actual current vibration signal. At the final step, this residual signal is considered a discriminative representation containing fault feature information and describing the current state of the system that is employed as an input to the DNN to accomplish the problem coupling blade rub-impact fault identification.

### 2.1. Data Collection

**Figure 2:**
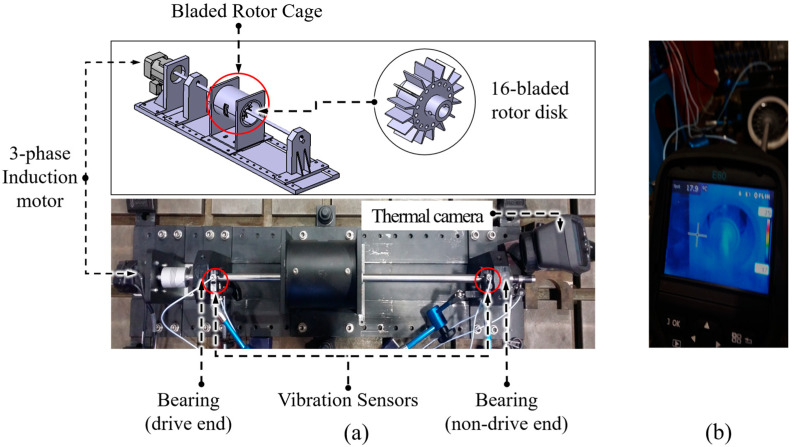
The test rig used for data collection: (**a**) the model and actual view of the rotor system; (**b**) the screen of the thermal camera used for validating the severity of the mechanical fault.

To investigate the capabilities of the proposed framework for blade rubbing fault identification, an experimental dataset was acquired in this study. The test rig used to collect rub-impact fault data of various severity levels is presented in Figure 2. Two vibration sensors installed at the different ends of the shaft were used to collect the data during the experiment. Each of the sensors has two channels for recording the displacements of the rotor in vertical and horizontal directions.

In this work, the coupling shaft imbalance and periodical local blade rub-impact fault has been simulated. Specifically, to create the interactions between rotor disk blades (the number of blades of the rotor disk is equal to 16) and rotor cage (i.e., blade rub-impact fault), shaft imbalance fault (<45°) has been first simulated by attaching additional weights to the rotor disk. The detection and evaluation of the severity levels of the fault being investigated have been done by a thermal camera mounted on the non-drive end of the rotor. Overall, 10 classes of signals were observed during data collection. Specifically, class #1 corresponds to 0 g of extra weight added to the shaft, which represents the normal operating state of the system. Classes #2, #3, and #4 correspond to 0.5, 1, and 1.5 g of extra weight added to the shaft. At this time, a shaft imbalance fault appeared in the testbed; however, despite the rotor imbalance, no contact between the blades and the stator was detected through the thermal camera. Classes #5 to #7 describe the first evidence that a coupling fault appeared in the system when shaft imbalance caused a slight blade rub fault with the 1.6, 1.7, and 1.8 g of additional weight added to the shaft, respectively. Classes #8 and #9 with 2 and 2.4 g of additional weight correspond to the coupling fault of shaft imbalance with an intensive blade rub, while class #10 with 2.8 g of extra weight attached to the rotor disk represented the coupling fault when the shaft imbalance led to a severe blade rub-impact fault condition.

The experiment was conducted under constant rotational speed equal to 2500 revolutions per minute (RPM). The vibration signals were collected at a sampling rate of 65.5 kHz. The duration of signal recordings for each signal class was 30 s. Then, for signal processing purposes, these signals were split into samples of 1 s in length. Thus, the total number of data instances acquired during the experiment was equal to 300 samples before cutting them into windows during signal resampling.

The main properties of the collected signal classes are summarized and presented in Table 1.

### 2.2. Signal Resampling

In general, deep learning-based approaches require datasets with a huge number of samples for efficient representation learning. However, it is not always possible and even expensive to collect huge datasets with the samples corresponding to faulty conditions of the system. Furthermore, when the artificial intelligence algorithms are applied to one-dimensional signals, the size of these input signals affects the architecture of the network (i.e., depth of the network, number of nodes, shape) as well as the time needed for learning these representations. To address these issues prior to creating the autoencoder-based nonlinear observer, in this work, we perform a resampling of the collected vibration signals corresponding to different states of the system into a series of windows such that each window has a length equal to the number of data points collected during one revolution of the shaft.

For vibration signal resampling, first, the number of revolutions completed in one second (RPS) should be computed by the formula as below:

(1) RPS=RPM/60,

where *RPM* is the rotational speed used during data recording.

Next, the time needed for one revolution (2) and the number of data points (3) collected during one revolution of the shaft can be obtained as shown below:

(2) $$TFOR=\frac{1}{RPS}\;,$$

(3) $$w\_length=f_{sampling}\times TFOR.$$

Here, TFOR stands for the time for one revolution expressed in seconds, w_length corresponds to the length of each window of the resampled signal expressed in a number of data points, and $f_{sampling}$ is the sampling frequency used during the data acquisition.

**Figure 3:**
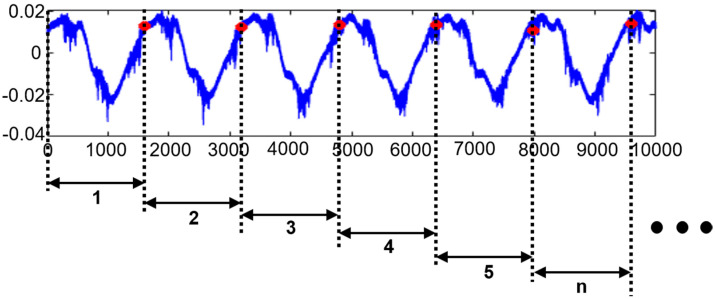
The vibration signal resampling process.

The computed parameters for resampling the signal into windows are as follows: RPS≈41.6, TFOR= 0.024, and w_length ≈1598, respectively. An example of signal resampling using the achieved resampling parameters is depicted in Figure 3.

### 2.3. Deep Undercomplete Denoising Autoencoder (DUDAE)-Based Nonlinear Function Approximation of the System and Residual Signal Generation

Autoencoders are a form of deep neural network that are widely used for problems where manifold learning is required. The most common tasks that are solved by autoencoders are feature learning [42], feature extraction [43], and feature selection [44]. However, since autoencoders are deep neural networks with a symmetric structure, they can successfully utilize the properties of neural networks to learn and discover complex nonlinear relations of the input data (i.e., nonlinear function approximation) and successfully utilize them for the input data reconstruction, which is the purpose of the autoencoder in this paper.

The simple undercomplete autoencoder consists mainly of three layers that are trained in an unsupervised manner. The first layer is called the input layer. It receives the input data and pushes it to the further layers. The hidden layer after the input layer with lower dimensionality is called a bottleneck layer. It is used to extract the latent coding, i.e., the high-level representative features of the input data. The dimensionality of the latent codes is equal to the number of nodes in the bottleneck layer. The last layer, called the output layer, is used to decode the obtained latent codes and reconstruct the original input data. In summary, the autoencoder performs two tasks: (1) it encodes the input data into the latent coding, and (2) it decodes the latent coding to reconstruct the original data. The operation of the autoencoder can be summarized as follows:

(4) $$\begin{array}{c} e:x\to F\\ d:F\to x\prime \\ e,d=\mathrm{argmin}{\left(x-x\prime \right)}^{2}\;\; \end{array}.$$

As mentioned above, the simple undercomplete autoencoder has only one hidden layer: the bottleneck layer. During the encoding stage, the autoencoder receives the input data x of the dimensions $R^{m}$ and nonlinearly maps the input data to the latent coding F with the dimensions $R^{n}.$ The encoding process can be presented as below:

(5) F=f(Wx+b),

where F is the latent coding, W represents the weight matrix, b stands for the bias, and f is a nonlinear activation function. The decoding process of the autoencoder is described by:

(6) $$\hat{x}=f\prime \left(W\prime F+b\prime \right).$$

Here, $\hat{x}$ is the reconstructed output that resembles the input data, W′ is the weight matrix, b′ stands for the bias, and f′ represents an activation function of the decoder, respectively.

To perform the training of the autoencoder, the mean squared error (MSE) loss function should be calculated between the original input data and the reconstructed data using the following equation:

(7) $$L\left(\theta \right)=\frac{1}{N}\sum_{n=1}^{N}{\left(x-\hat{x}\right)}^{2},$$

where L stands for the MSE loss function, θ is a set of model parameters, and N is the dimensionality of the input data, i.e., the number of nodes in the input layer of the autoencoder.

In this paper, the DUDAE is utilized to approximate the nonlinear function of the normal state of the rotor system. The detailed architecture of this autoencoder is presented in Table 2. Unlike the simple three-layer undercomplete autoencoder, the proposed DUDAE is a deep autoencoder (emphasized by the first ‘D’ in the abbreviation) that has more than one hidden layer, as can be seen in the table. However, the basic idea described in Equations (4)–(7) pertains to the DUDAE, with the only difference being that during the encoding and decoding phases, more nonlinear data transformations are done concerning the increased number of hidden layers. From the same table, it can be seen that the size of the encoding layers is smaller than that of the input layer, which means that the structure of the proposed autoencoder is “undercomplete” (highlighted by the “U” in the abbreviation). This is needed to force the autoencoder to learn a more compact representation (i.e., nonlinear function) from the input data. To increase the tolerance to the noise of the autoencoder used for approximating the nonlinear function of the normal operating state of the system, the dropout [45], with a rate equal to 0.1, is added to the input layer in which the input signals are received. This makes the proposed autoencoder belong to a type of denoising autoencoders (this property is expressed as the second ‘D’ in the abbreviation).

As the activation function for the hidden and output layers of the DUDAE, the scaled exponential linear units (SELU) function is chosen in this paper. There are a few main reasons of employing this activation function: (1) the input vibration signals collected by the sensors contain both the positive and negative values, hence a possibly non-saturating activation function that supports these types of inputs is needed, (2) the specific formulation of the SELU activation prevents the vanishing gradient problem that may be faced in deep architectures, as well as avoids the situations when the neuron can die during training, and (3) the SELU activation function speeds up the training process and convergence of the deep neural network due to its normalization properties [46]. The formulation of the SELU activation function is shown in Equation (8):

(8) $$f_{selu}\left(x\right)=\lambda \{\begin{array}{ll} \alpha e^{x}-\alpha & x\leq 0\\ x & x>0 \end{array},\;\;$$

where λ≈1.05 and α≈1.6731 are the coefficients predetermined by the inventors of SELU activation [42].

Glorot uniform weight initialization [47] was chosen as the initialization strategy of the weights in the hidden layers of the proposed deep undercomplete denoising autoencoder.

As the optimization algorithm for training the deep denoising undercomplete autoencoder to estimate the nonlinear function of the normal system state using backpropagation, the RMSProp optimizer [48], a widely used variant of stochastic gradient descent for training autoencoders, is applied in this paper. The equation of this optimization algorithm can be presented as follows:

(9) $$\begin{array}{c} s\leftarrow \gamma s+\left(1-\gamma \right)\nabla_{\theta }L\left(\theta \right)⊗\nabla_{\theta }L\left(\theta \right)\\ \theta \leftarrow \theta -\xi \nabla_{\theta }L\left(\theta \right)⊘\sqrt{s+\varepsilon } \end{array}.$$

Here, s is a vector containing the squares of the loss function gradients; γ stands for the rate of decay (γ=0.9);$\;\nabla_{\theta }L\left(\theta \right)$ represents the gradient of the loss function (MSE in this case) with the respect to the parameters of the deep learning model, θ; ξ is the notation for the learning rate that was assigned to be equal to 0.001; ε is the coefficient needed to prevent zero division (ξ =$\;10^{-7}$ in this paper); and ⊗ and ⊘ are the operators of element-wise multiplication and division, respectively.

### 2.4. Residual Signal Generation

The main purpose of the autoencoder (DUDAE) in this paper is to learn the nonlinear function of the system under normal operating conditions. Once the training is completed, this trained model is used to give its estimate of the current system state by attempting to reconstruct the signal previously unseen during the training (i.e., a signal corresponding to the unknown state of the system). Next, the residual signals are generated as a difference signal between the real unknown vibration signal and the estimate of this signal delivered by the DUDAE. These residual signals are used at the next step as the input for the DNN to perform fault identification, and can be computed as below:

(10) $$r_{\hat{x}}\left(n\right)=x\left(n\right)-\hat{x}\left(n\right),$$

where $r_{\hat{x}}\left(n\right)$ is the residual signal, x(n) stands for the original vibration signal in the time domain, and $\hat{x}\left(n\right)$ is the signal reconstructed by the autoencoder using the latent coding learned while training on signals corresponding to the normal operating state of the system (i.e., when no imbalance and no blade rub fault are observed).

The purpose of computing the residual signals is as follows. Since the DUDAE is trained using only the data collected under normal system operating conditions, it learns how to reconstruct this data by using the learned nonlinear function, i.e., latent coding. However, it cannot accurately reconstruct the data that have not been used during the training process. That is, if the DUDAE is applied to reconstruct the signals not observed during training and that significantly deviate from the signals corresponding to the normal system state, it will inevitably lead to a reconstruction error. Furthermore, when a shaft imbalance or a coupling imbalance and blade rub fault appears in the system, the values of statistical parameters of the vibration signals increase with the increase of their amplitude. This means that errors between the real signals corresponding to abnormal conditions of the system and the ones estimated by the DUDAE will increase, too. This allows for detection of the current state of the system and residual signals computed by Equation (10) can be used as discriminative features to perform fault identification of coupling blade rub faults of various intensity levels.

### 2.5. Fault Identification Using Residual Signals and the DNN

Despite the fact that the DNN is a variation of the conventional ANN, which was first introduced a long time ago, due to the higher dimensionality and the number of hidden layers, it became one of the most powerful and widely applied decision-making algorithms for a huge variety of problems. Furthermore, DNNs became the main core of recent trends in the field of artificial intelligence algorithms, such as deep representation learning.

The general DNN architecture resembles the architecture of an ANN and consists of an input, output, and a sequence of hidden layers. The generalized formula of the $m^{th}$ hidden layer operation can be summarized as follows:

(11) $$x_{m}=f\left(W_{m}x_{m-1}+b_{m}\right),$$

where $x_{m}$ is the output of the $m^{th}$ hidden layer after applying the nonlinear activation function f; $x_{m-1}$ is the output of the previous hidden layer after application of the activation function; and $W_{m}$ and $b_{m}$ are the weight matrix and bias vector of the $m^{th}$ hidden layer, respectively.

In this paper, the DNN is used to perform the task of blade rub-impact fault identification using the residual signals computed using Equation (10). The exact architecture of the DNN used for fault identification is presented in Table 3. As can be seen from the table, the architecture of the proposed DNN is similar to the encoder part of the DUDAE described in Section 2.3. However, there are two differences that are discussed below.

The first is the way the dropout regularization has been applied. Unlike the autoencoder, where the dropout was applied only to the input layer to increase its robustness to the noise in the data, in the DNN, it is used for fault identification, and a dropout rate of 0.1 is applied to hidden layers #2, #3, #4, and #5 to avoid overfitting of the data. If the DNN overfits the training data, it might fail to generalize the validation and testing data (the data unseen during the training process), which will lead to a decrease in the fault classification performance. It cannot be seen from the table, but along with dropout regularization, an early stopping procedure is applied during the training of the DNN to reduce the chance of overfitting. The idea of early stopping is to interrupt the training process once the validation error stops decreasing or starts increasing with some tolerance level during a defined number of epochs.

The second difference is an activation function of the output layer. To solve a multiclass classification problem, SoftMax activation is employed in the output layer of the DNN. The SoftMax activation function is given as follows:

(12) $${\hat{P}}_{k}=exp\left(s_{k}\left(x\right)\right)/\sum_{i=1}^{K}exp\left(s_{i}\left(x\right)\right),$$

where K is the total number of classes and s(x) is a vector containing the scores of each class for the specific data instance x. The input data instance is assigned to the class with the highest estimated probability ${\hat{P}}_{k}$ (i.e., the class with the highest computed score for this sample).

To train the DNN to perform blade rub fault identification using the residual signals, the categorical cross-entropy loss function is used with the outputs of the SoftMax activation of the output layer to perform decision making about the state of the system. The categorical cross-entropy loss can be represented as below:

(13) $$Loss\left(\theta \right)=-\frac{1}{n}\overset{n}{\underset{i=1}{\sum }}\overset{K}{\underset{k=1}{\sum }}y_{k}^{i}\log\left({\hat{P}}_{k}\right),$$

where θ is the set of model parameters and $y_{k}^{i}$ and ${\hat{P}}_{k}$ are the target and estimated probabilities that the $i^{th}$ data instance belongs to the class k, respectively. The same optimization algorithm used for training the autoencoder, RMSProp (Section 2.3, Equation (9)), is used for training the DNN by computing the gradients of the categorical cross-entropy loss function with respect to model parameter θ.

The remaining parameters of the machine learning model, such as the weight initialization algorithm, the learning rate of the optimization algorithm, and other parameters of the network remain the same, as described in Section 2.3.

## 3. Experimental Results and Discussion

### 3.1. Training, Validation, and Testing Data Configuration

After signal resampling, described in Section 2.2, the new dataset consisted of 12,300 time-domain resampled vibration signals in total (1230 resampled signals for each system condition observed during data collection). To investigate the fault identification capabilities of the proposed approach, the two experimental datasets were constructed.

The first dataset consisted of all the resampled time-domain vibration signals corresponding to the normal state of the system (1230 resampled signals), i.e., when neither imbalance nor coupling imbalance and blade rub faults were observed (this dataset is further referred to as dataset #1). This dataset is needed to train the DUDAE to reconstruct the input data using the learned latent coding and to derive the residual signals that are further used for fault identification by the DNN. For training the DUDAE, dataset #1 was randomly divided into training and validation subsets at a rate of 8:2. Thus, 984 resampled signals corresponding to normal system conditions were used as a training subset for the DUDAE, whereas the remaining 246 signals comprised the validation subset used to measure validation error.

Once the autoencoder was trained, it was used to generate the residual signals using the whole 12,300 original resampled vibration signals. The data were used further to accomplish the task of fault diagnosis. For this, the dataset of residual signals (further referred to as dataset #2) was first randomly split into training and testing subsets at a ratio of 8:2. Then, the obtained training subset was randomly split again at the ratio 8:2 to get a validation subset. Thus, the obtained training subset from dataset #2 consisted of 7872 residual signals, the validation subset contained 1968 samples, and the remaining 2460 residual signals previously unseen by the DNN were used as a testing subset for evaluating the fault diagnosis capabilities of the proposed framework.

To ensure the reliability of the proposed methodology and exclude the effect of randomness, the experiments for the proposed and referenced methods will be performed 10 times with different training, validation, and testing subsets randomly sampled at each trial.

### 3.2. Training the DUDAE–DNN Model

Before validating the capabilities of the proposed framework to identify blade rub-impact faults of various intensity levels, the modules of the proposed framework, the DUDAE and the DNN, should be trained. Furthermore, they should be trained in a pipeline (i.e., sequential order). Thus, first, the training and validation subsets of dataset #1 containing the time-domain resampled vibration signals corresponding to the normal condition are used to train the DUDAE. Next, the training and validation subsets of dataset #2 (consisting of residual signals obtained after training the DUDAE) are utilized to train the DNN to perform fault diagnosis. For training both parts of the model, data batches with 64 data samples each were utilized. Initially, the number of training epochs for the DNN model was assigned to be equal to 1000. However, the early stopping algorithm was applied during the training that stops the learning process once the validation accuracy stops improving and restores the model parameters that demonstrated the highest fault classification accuracy on the validation subset.

**Figure 4:**
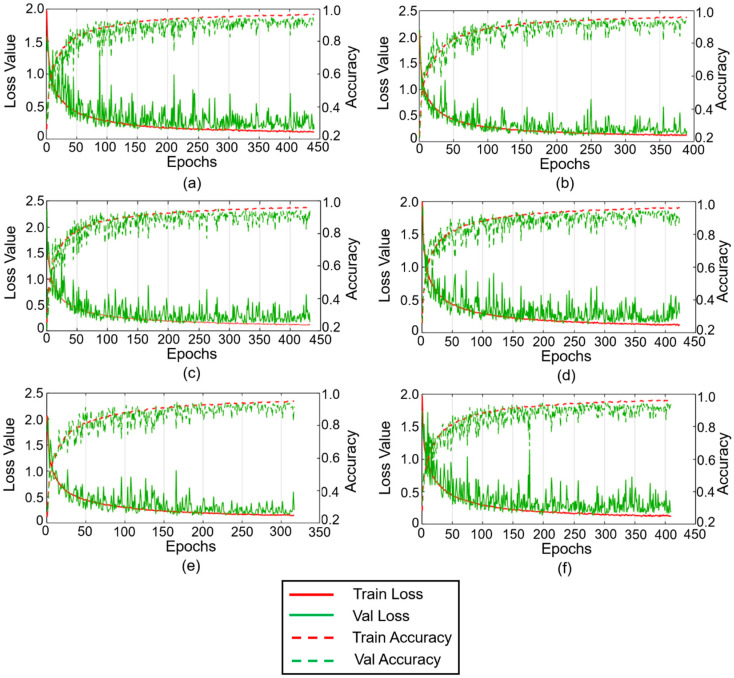
The convergence curves obtained after training the DNN on residual signals computed after the DUDAE was trained during (**a**) 100 epochs, (**b**) 200 epochs, (**c**) 300 epochs, (**d**) 400 epochs, (**e**) 500 epochs, and (**f**) 600 epochs, respectively.

In Figure 4, the analysis of dependence between the number of training epochs of the DUDAE model and its influence on the validation classification accuracy of the DNN is presented.

From this figure, it can be seen that in general, the increase in the number of training epochs for the DUDAE model leads to a decrease in the number of training epochs for the DNN model (the early stopping technique stops training earlier, since no improvement on the validation subset has been observed for a certain number of epochs). At the same time, for all of the cases presented in Figure 4, it can be observed that at the moment when the training was stopped, the validation accuracy curve saturates and slightly oscillates around an accuracy of 94–95%. However, when we applied the best model parameters saved during the DNN training in each experiment, it appeared that the model trained on residual signals obtained after training the DUDAE during 600 epochs reached 95.5% accuracy on the validation subset. The other models demonstrated slightly worse performance: 95.33%, 95.2%, 95.4%, 95.4%, and 95.1% for 100, 200, 300, 400, and 500 training epochs of the DUDAE, respectively. Overall, the number of training epochs of the DUDAE did not significantly affect the fault classification performance of the DNN. However, considering that the validation accuracy of the DNN was slightly higher when the DUDAE was trained over 600 epochs, it was decided to keep this number of training epochs for the DUDAE while the number of training epochs of the DNN was left under full control of the early stopping algorithm.

**Figure 5:**
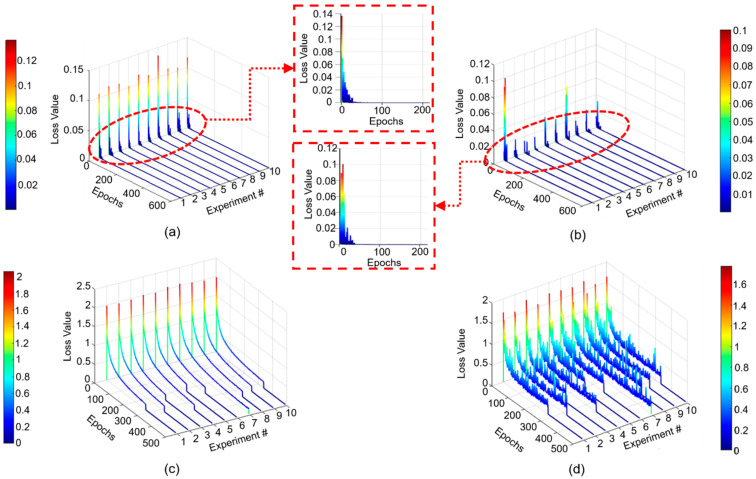
The training and validation loss curves obtained during ten experimental trials by the proposed technique: (**a**) training loss curves of DUDAE, (**b**) validation loss curves of DUDAE, (**c**) training loss curves of DNN, and (**d**) validation loss curves of DNN, respectively.

Once the training epoch number of DUDAE and training scenario for decision-maker (DNN) are fixed, we repeat the training–validation procedure 10 times to observe the behavior of the training–validation loss curves and generalize the conclusions on the convergence of the proposed methodology. The training and validation loss curves obtained during 10 experiments are presented in Figure 5.

The training and validation curves corresponding to DUDAE are demonstrated in Figure 5a,b. From these figures, it can be seen that the values of loss functions during ten experiments first demonstrated a sharp descent during the first 40 epochs of training and then continued decreasing toward zero steadily. Despite in all experimental trials DUDAE having been trained during 600 epochs, from Figure 5c,d, we can observe that the training process of DNN has been stopped by an early stopping algorithm at different moments before 400 epochs in all trials except for in experiment #7, where the training of DNN has lasted for 488 epochs (the longest result). From Figure 5d and its color bar, it can be concluded that in all experimental trials, the validation loss curves of DNN demonstrated similar descending patterns, and at the moment when the training procedure was stopped, they were oscillating around the value of 0.2.

Overall, it can be concluded that the proposed methodology demonstrates repeatable results in terms of convergence under various training and validation subset permutations. However, it can be also seen that there is an open direction for improvement of the part related to the decision making in the proposed framework because despite a good convergence of DUDAE under various data permutations, the loss functions of DNN saturated at a certain level without moving closer to zero.

### 3.3. Residual Signal Analysis

**Figure 6:**
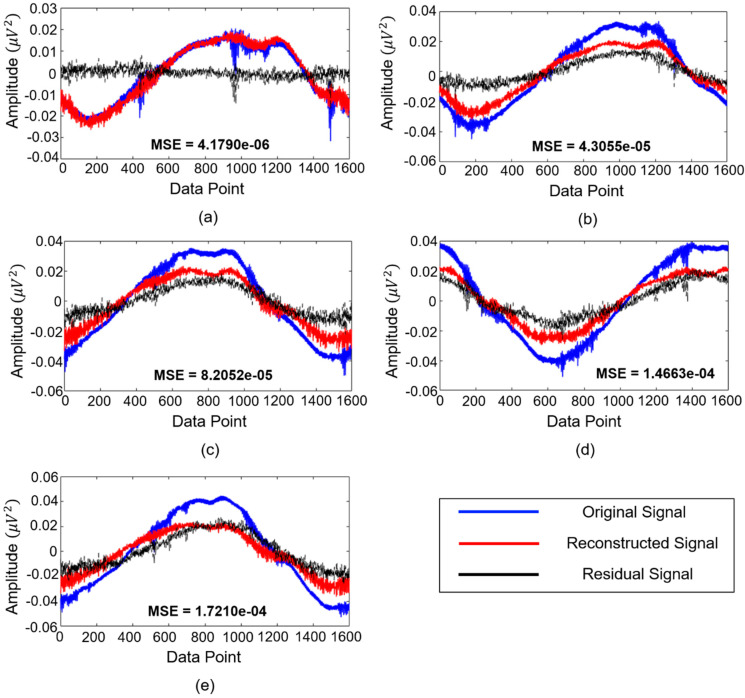
The original, reconstructed, and residual signal examples corresponding to signal classes of (**a**) normal operating condition, (**b**) 1.5 g shaft imbalance condition, (**c**) 1.7 g shaft imbalance + slight blade-rub fault condition, (**d**) 2.4 g shaft imbalance + intensive blade rub-fault, and (**e**) 2.8 g shaft imbalance + severe blade rub fault, respectively.

In this subsection, the analysis of residual signals obtained after the DUDAE was trained on signals corresponding to the normal system state is provided. From the previous subsection, it was concluded that when the number of training epochs of the DUDAE is equal to 600, the obtained residual signals that are used as input to the DNN for decision making on the state of the system lead to the highest classification accuracy on the validation dataset. The main point of this is that the well-trained DUDAE delivers residual signals of a small magnitude oscillating around zero (i.e., small reconstruction error) for the signals that correspond to the normal state of the system or for the signals that resemble those signals. On the other hand, when the imbalance and blade rub-impact fault appear in the system, the vibration signals start deviating from the ones corresponding to a normal operating state. Hence, with the increase of rub-impact fault intensity, the reconstruction error increases as well, which leads to residual signals of higher magnitudes and higher deviations from zero. The examples of residual signals computed after the trained DUDAE for different states of the system are depicted in Figure 6.

As can be seen from this figure, the magnitudes of residual signals and their shapes change with the progression of the fault. Furthermore, it can be seen that MSE values computed between the original and reconstructed signals also increase when the signals in the input of the trained DUDAE deviate significantly from the signals corresponding to the normal system condition when neither shaft imbalance nor blade rub faults was observed.

**Figure 7:**
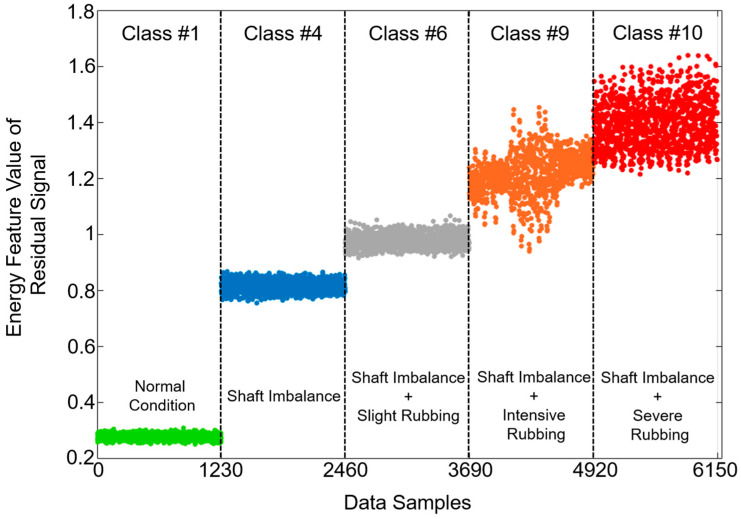
The energy feature parameter used for characterizing the residual signals obtained by proposed methodology for the signal classes under normal operating condition, 1.5 g shaft imbalance condition, 1.7 g shaft imbalance + slight blade-rub fault condition, 2.4 g shaft imbalance + intensive blade rub-fault, and 2.8 g shaft imbalance + severe blade rub fault, respectively.

Figure 7 illustrates the energy of residual signals generated by the proposed methodology for five signal classes, namely normal system condition (class #1), shaft imbalance fault (class #4), shaft imbalance + slight rubbing fault (class #6), shaft imbalance + intensive rubbing fault (class #9), and shaft imbalance + severe rubbing fault (class #10), respectively. The signal groups presented in Figure 7 are the same as the ones demonstrated in Figure 6 for the sake of consistency. In the proposed methodology, DUDAE extracts the function of the dynamic behavior of the normal signal (the rotor system is under the normal operating condition when no faults are observed) during its training. However, in abnormal conditions of the system, the behavior of the signal is utterly different from its behavior in the normal state of the system. Regarding Figure 7, it can be seen that the accuracy of the dynamic behavior estimation for the signals belonging to different classes is satisfactory, especially for class #1. The reason for this observation is that the residual signal itself is a type of error signal that is computed between the actual vibration signal and one estimated by DUDAE. That is, since the DUDAE has been trained on signals belonging to normal conditions, it is capable of accurately estimating the unknown signals when their dynamic behavior is close to the ones it has been learned on. Furthermore, it can be seen from Figure 6 and Figure 7 that when we use DUDAE to estimate the unknown signal dynamic behavior that drastically differs from ones collected under normal operating conditions, the estimation error (residual signal) between the actual and estimated signal is increasing. In Figure 6, this difference can be observed in a deviation of residual signals from zero-mean along with the increasing value of the MSE metric, while in Figure 7, this difference is characterized with growing values of energy features extracted from those residual signals. Based on the energies of the residual signals presented in Figure 7, it can be concluded that the obtained residual signals are sensitive to the degradation of the system, which means that these residual signals can be used as discriminative features itself for fault classification or for the feature extraction in conjunction with feature-based machine learning classifiers for diagnosing faults. Thus, the more discriminative the residual signals are, the easier it is for the classifier to perform fault identification. However, some overlap can be observed when the intensity of rub fault increases, such as in classes #9 and #10. Therefore, to improve the potential fault classification accuracy, the DNN with the residual signals as input features is recommended in this work instead of conventional amplitude-based statistical feature extraction and fault classification schemes, the performance of which can be affected by the overlap of the extracted feature parameters.

### 3.4. Fault Identification Performance

To evaluate the fault identification capabilities of the proposed framework, we compare it with two counterpart methods. Since the proposed model is a pipeline process containing two steps, nonlinear function approximation of the system state and decision making, for fair comparison, it is reasonable to fix the decision-making approach (i.e., DNN) and vary the methods at the first step to observe whether the proposed pipeline influences the fault identification abilities or not. The first method used for the comparison is directly applying the DNN to resampled signals in the time domain (further referred to as RAW+DNN). This approach allows us to investigate the improvement in classification performance of the proposed method where nonlinear function approximation by the DUDAE is utilized in comparison to when no function approximation is used. In the second approach used for the comparison, we are utilizing a widely used state-of-the-art linear observation method from the field of control theory, autoregressive with external input ARX–Laguerre proportional–integral observer (PIO) (ARXLPIO) [21], for estimating the nonlinear blade rub-impact fault signals. The residual signals computed as the difference between the original raw signals and ones estimated by ARXLPIO are used as the inputs to the DNN to accomplish the task of fault diagnosis. This method will be further referred to as ARXLPIO+DNN. The architecture of the DNN employed in the comparison approaches matches the one used in the proposed DUDAE+DNN model. Note, in this comparison, we are not using the modern control theory algorithms, such as nonlinear observation techniques. The main reason for this, as was discussed in the introduction part of this manuscript, is the complexity of the design process of these approaches in a real industrial environment as well as the need to re-design the nonlinear observation technique whenever the system changes. Additionally, to investigate the quality of obtained residual signals and compare the performance of different types of techniques for residual signal classification (i.e., fault identification), the two additional techniques that include residual signal characterization with feature parameter and classification are included in this experiment. One method represents the characterization of residual signals with the energy feature parameter and decision tree machine learning algorithm, as has been proposed in [21] (further referred to as RS+EN+DT). As the second approach used for residual signal characterization and classification, the combination of the SVM machine learning classifier, as one of the most popular classification algorithms, is applied to the energy features extracted from the residual signal [49] (further referred to as RS+EN+SVM).

The fault classification performance for the methods mentioned above is evaluated using the widely micro-averaged forms of widely used metrics [50], such as micro-averaged recall ($Rec_{\mu }$), micro-averaged precision ($Prec_{\mu }$), micro-averaged F1-score ($F1_{\mu }$), and total fault identification accuracy (FIA). It is decided to use the micro-averaged versions of these metrics to address the possible deviations in the numbers of data samples presented in each class in the testing subsets due to the random sampling procedure applied at each trial of the experiment. These metrics are expressed as follows:

(14) $$Rec_{\mu }=\frac{\sum_{k=1}^{K}TP_{k}}{\sum_{k=1}^{K}\left(TP_{k}+FN_{k}\right)}\times 100;$$

(15) $$Prec_{\mu }=\frac{\sum_{k=1}^{K}TP_{k}}{\sum_{k=1}^{K}\left(TP_{k}+FP_{k}\right)}\times 100;$$

(16) $$F1_{\mu }=2\times \left(Prec_{\mu }\times Rec_{\mu }\right)\times 100/\left(Prec_{\mu }+Rec_{\mu }\right);$$

(17) $$FIA=\frac{\sum_{k}^{K}TPV_{k}}{N}\times 100$$

Here, $TP_{k}$,$\;FP_{k}$, and $FN_{k}$ are the true-positive, false-positive, and false-negative values computed for the data instances of the class k, respectively; N is the total number of data samples in the datasets used for the experiment, and K is the total number of signal classes presented in the datasets. The experimental results expressed in these metrics averaged over 10 experiments are tabulated in Table 4.

**Figure 8:**
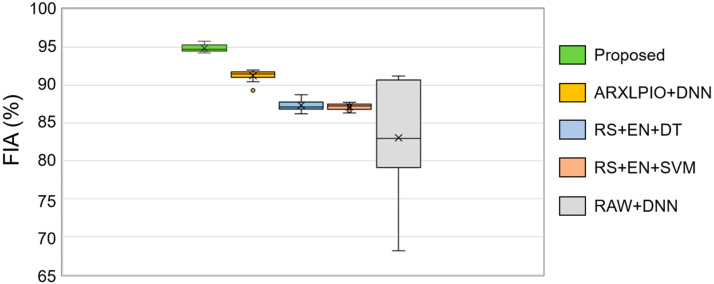
The boxplots demonstrating the statistics of the FIA metric over 10 experiments.

As can be seen from the table, the proposed framework outperformed the counterpart methods with the highest average FIA of 94.8%. The counterpart approaches, ARXLPIO+DNN, RS+EN+DT, RS+EN+SVM, and RAW+DNN, achieved FIAs of 91.2%, 87.3%, 87.1%, and 83.0%, respectively. More details regarding fault classification accuracies can be observed in Figure 7 where the boxplots with distributions of accuracy values obtained during 10 experiments are presented. The black cross in the boxes belonging to different methods in Figure 8 corresponds to the average classification accuracy values presented in Table 4.

As can be seen from the boxplots demonstrated in Figure 8, the classification accuracy values did not deviate significantly from the mean and median values during the experiments for the proposed method, which ensures the repeatability of the results. For ARXLPIO+DNN, it can be seen that the deviation of the accuracy values is also not very significant, with outliers not laying far from the box; however, all the accuracy values are distributed lower than the results of the proposed technique. Unlike the proposed method where artificial intelligence-based system identification has been used and ARXLPIO+DNN where the linear observer has been utilized, we can see that the box corresponding to the RAW+DNN method is wider with a long whisker laying toward the outlier of 68.1%. From this figure, it can be concluded that the proposed DUDAE used for nonlinear function approximation and the ARXLPIO observation technique both can improve the fault diagnosis stability; however, the DUDAE helps to increase the average classification performance when applied to a nonlinear rubbing signal in comparison with a linear observation technique. Additionally, both RS+EN+DT and RS+EN+SVM techniques, where the residual signals provided by DUDAE have been characterized by an energy feature parameter, provided relatively high results in terms of average FIA. Furthermore, these methods demonstrated small deviations of the FIA metric from its mean value during 10 experiments, which also shows that even less powerful classifiers in comparison with DNN are capable of yielding stable results under different training–testing data permutations when applied to the features extracted from residual signals delivered by the proposed technique. The results of RS+EN+DT and RS+EN+SVM also speak for the advantage of the proposed technique and highlight the importance of the quality of residual signals. If the residuals are of high quality, various algorithms for classifying these residual signals can be used without significant performance degradation.

**Figure 9:**
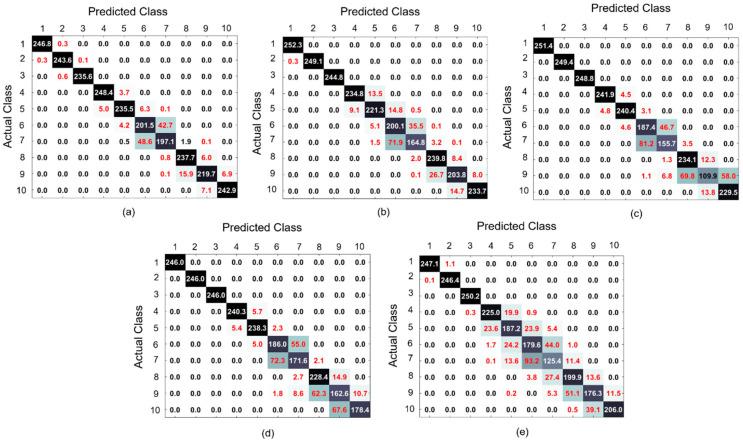
The confusion matrices obtained for (**a**) proposed, (**b**) autoregressive with external input ARX–Laguerre proportional–integral observer (PIO) (ARXLPIO)+DNN, (**c**) the characterization of residual signals with the energy feature parameter and decision tree machine learning algorithm (RS+EN+DT), (**d**) combination of the SVM machine learning classifier with the energy features extracted from the residual signal (RS+EN+SVM), and (**e**) directly applying the DNN to resampled signals in the time domain (RAW+DNN), respectively. The presented results are averaged over 10 experiments.

Figure 9 presents the confusion matrices averaged over 10 experiments to provide more details on the fault diagnosis performance. From this figure, it can be seen that the proposed technique demonstrated the lowest numbers of misclassifications in conditions where the nonlinearity of the rotor system increases in comparison with referenced techniques, especially, with the RAW+DNN where the DNN has been applied directly to vibration signals. These conditions corresponded to the appearance of coupling imbalance and slight blade rub-impact faults (classes #5–7) and imbalance with intensive blade rubbing faults (classes #8 and #9). From Figure 9c,d, it can be observed that despite demonstrating relatively good results of fault classification when different feature-based machine learning classifiers are applied to the energy feature parameters extracted from the residual signals provided by the proposed technique, those classification results can be still improved by using more powerful approaches for decision making that might extract discriminative features autonomously or utilize the residual signal itself as a feature, such as DNN.

The main reason that the ARXLPIO+DNN method shows degraded performance in comparison with the proposed methodology is in the nature of the ARXLPIO method. When a linear observation technique, such as ARXLPIO, is applied to approximating the nonlinear function of the system state when dealing with nonlinear and nonstationary signals, it inevitably leads to the appearance of estimation errors. This is because the uncertainty term of the nonlinear and nonstationary signal (i.e., blade rub-impact fault signal) modeling cannot be estimated properly by the linear observation technique. Despite the fact that the residual signals obtained after the ARXLPIO observation technique appear to be more discriminative as features in comparison with the original raw time-domain signals, the degradation of classification performance in comparison with the nonlinear observation techniques from the field of control theory or the artificial intelligence-based techniques is expected.

The RAW+DNN method demonstrated the lowest FIA in comparison to other techniques presented in Table 4. In this approach, the DNN utilized the raw resampled time-domain vibration signals as the inputs to perform the task of fault identification. It mainly demonstrated lower accuracy in comparison with the proposed approach due to the complexity of the blade rub-impact fault signal. Due to its non-stationarity, the statistical properties of time-domain samples may vary over time even when they belong to the same signal class, which leads to the problem that time-domain vibration signal patterns are not discriminative enough and may lead to the failure of the DNN to adjust its weights during training to reach a good level of generalization.

Overall, it can be concluded that the proposed data-driven framework consisting of the DUDAE-DNN model is suitable for diagnosing blade rub-impact faults of various intensity levels with high fault classification accuracy in comparison with the other referenced methods. From the experimental results, it can be seen that the application of the DUDAE for approximating the nonlinear function of the nonlinear rotor system state improves the fault diagnosis capabilities of the DNN in comparison with the state-of-the-art linear observation techniques frequently used in industry and the situations when no signal observation is used. Another important advantage of the proposed methodology is that its structure is pipeline-shaped, which supports modifications of the current architecture as well as applicability to other systems, since the residual signals used as features in this study are generated based on the ideas of system identification. However, from the results, it also can be seen that the proposed methodology for diagnosing coupling blade rub-impact faults still should be improved to increase its classification performance when dealing with vibration signals corresponding to the increasing nonlinearity of the rotor system. Furthermore, to accomplish a comprehensive investigation of the robustness and reliability, it is important to test the proposed methodology on the datasets with varying operating conditions, such as varying rotating speed and varying load.

## 4. Conclusions

In this paper, a novel method for diagnosing complex coupling faults consisting of shaft imbalance and blade rub-impact faults of different severity levels is introduced. In the proposed fault diagnosis technique, the input time-domain vibration signals are first resampled concerning the fundamental frequency of the rotating machine. Then, the nonlinear function approximation of the system state under normal operating conditions is accomplished by training the deep undercomplete denoising autoencoder on the resampled signals corresponding to the state of the system when neither imbalance nor blade rub-impact faults were observed. Next, the residual signals are computed as a difference between the original resampled time-domain signals and their estimates by the autoencoder. Finally, these residual signals were used as the inputs to the deep neural network to perform the decision making about the current state of the rotor system. The series of experiments show that the proposed fault diagnosis model demonstrated stable convergence behavior under different training–testing data permutations and outperformed other methods used for the comparison in terms of the micro-averaged performance metrics. In future work, we will focus on the improvement of the robustness and reliability of the proposed methodology. The possible directions for the improvement are the discovering and application of more complex architectures of an autoencoder to improve the quality of nonlinear function approximation and deep neural network to improve the decision-making procedure that will lead to better classification of the vibration signals with the induced nonstationary. Furthermore, the problem of varying operating conditions should be considered, and the proposed technique should be validated using the datasets containing other mechanical faults with changing operating conditions.

## Figures and Tables

**Table 1:** The main properties of the collected signal classes.

	Class #									
	1	2	3	4	5	6	7	8	9	10
Weight (g)	0.0	0.5	1.0	1.5	1.6	1.7	1.8	2.0	2.4	2.8
Sample #	30	30	30	30	30	30	30	30	30	30
System State	Norm.	Shaft Imbalance			Shaft Imbalance Slight Rub			Shaft Imbalance Intensive Rub		Shaft Imbalance Severe Rub

**Table 2:** The architecture of the proposed deep undercomplete denoising autoencoder (DUDAE).

Layer #	Type of Layer (Purpose)	Node #	Activation	Dropout
#1	Input (Encoder)	1598	-	0.1
#2	Hidden (Encoder)	790	SELU	-
#3	Hidden (Encoder)	395	SELU	-
#4	Hidden (Encoder)	128	SELU	-
#5	Hidden (Encoder)	64	SELU	-
#6	Hidden (Encoder/Decoder)	32	SELU	-
#7	Hidden (Decoder)	64	SELU	-
#8	Hidden (Decoder)	128	SELU	-
#9	Hidden (Decoder)	395	SELU	-
#10	Hidden (Decoder)	790	SELU	-
#11	Output (Decoder)	1598	SELU	-

**Table 3:** The architecture of the deep neural network (DNN) used for fault identification.

Layer #	Type of Layer	Node #	Activation	Dropout
#1	Input	1598	SELU	-
#2	Hidden	790	SELU	0.1
#3	Hidden	395	SELU	0.1
#4	Hidden	128	SELU	0.1
#5	Hidden	64	SELU	0.1
#6	Hidden	32	SELU	-
#7	Output	4	SoftMax	-

**Table 4:** The experimental results expressed in terms of $Rec_{\mu }$, $Prec_{\mu }$, $F1_{\mu }$, and fault identification accuracy (FIA) averaged over 10 experiments.

Methods	Metrics			
	$Rec_{\mu }\;(\%)$	$Prec_{\mu }\;(\%)$	$F1_{\mu }\;(\%)$	FIA (%)
Proposed	94.85	94.85	94.85	94.8
ARXLPIO+DNN	91.24	91.24	91.24	91.2
RS+EN+DT	87.33	87.33	87.33	87.3
RS+EN+SVM	87.13	87.13	87.13	87.1
RAW+DNN	83.05	83.05	83.05	83.0

## References

[1] Zhang Y., Wen B., Leung A.Y.T. (2002). Reliability Analysis for Rotor Rubbing. J. Vib. Acoust..

[2] Madhavan S., Jain R., Sujatha C., Sekhar A.S. (2014). Vibration based damage detection of rotor blades in a gas turbine engine. Eng. Fail. Anal..

[3] Willsch M., Bosselmann T., Theune N.M. New approaches for the monitoring of gas turbine blades and vanes. Proceedings of the IEEE Sensors.

[4] Mathioudakis K., Papathanasiou A., Loukis E., Papailiou K. (1991). Fast Response Wall Pressure Measurement as a Means of Gas Turbine Blade Fault Identification. J. Eng. Gas Turbines Power.

[5] Kim K.M., Park J.S., Lee D.H., Lee T.W., Cho H.H. (2011). Analysis of conjugated heat transfer, stress and failure in a gas turbine blade with circular cooling passages. Eng. Fail. Anal..

[6] Choy F.K., Padovan J. (1987). Non-linear transient analysis of rotor-casing rub events. J. Sound Vib..

[7] Chu F., Lu W. (2005). Experimental observation of nonlinear vibrations in a rub-impact rotor system. J. Sound Vib..

[8] Rubio E., Jáuregui J.C. (2011). Time-Frequency Analysis for Rotor-Rubbing Diagnosis. Advances in Vibration Analysis Research.

[9] Chandra N.H., Sekhar A.S. (2016). Fault detection in rotor bearing systems using time frequency techniques. Mech. Syst. Signal Process..

[10] Cheng J., Yu D., Tang J., Yang Y. (2009). Local rub-impact fault diagnosis of the rotor systems based on EMD. Mech. Mach. Theory.

[11] Prosvirin A.E., Islam M.M.M., Kim J.-M. (2019). An Improved Algorithm for Selecting IMF Components in Ensemble Empirical Mode Decomposition for Domain of Rub-Impact Fault Diagnosis. IEEE Access.

[12] Patel T.H., Darpe A.K. (2009). Coupled bending-torsional vibration analysis of rotor with rub and crack. J. Sound Vib..

[13] Zhihao J., Shangwei J., Wen J., Bangchun W. (2009). Rubbing Fault Diagnosis of Rotary Machinery Based on Wavelet and Support Vector Machine.

[14] Roy S.D., Shome S.K., Laha S.K. Impact of wavelets and filter on vibration-based mechanical rub detection using Neural Networks. Proceedings of the 2014 Annual IEEE India Conference (INDICON).

[15] Wan F., Xu Q., Li S. (2004). Vibration analysis of cracked rotor sliding bearing system with rotor–stator rubbing by harmonic wavelet transform. J. Sound Vib..

[16] Lei Y., Lin J., He Z., Zuo M.J. (2013). A review on empirical mode decomposition in fault diagnosis of rotating machinery. Mech. Syst. Signal Process..

[17] Tse P.W., Yang W., Tam H.Y. (2004). Machine fault diagnosis through an effective exact wavelet analysis. J. Sound Vib..

[18] Bessous N., Zouzou S.E., Bentrah W., Sbaa S., Sahraoui M. (2018). Diagnosis of bearing defects in induction motors using discrete wavelet transform. Int. J. Syst. Assur. Eng. Manag..

[19] Hasan M., Kim J.-M. (2019). Fault Detection of a Spherical Tank Using a Genetic Algorithm-Based Hybrid Feature Pool and k-Nearest Neighbor Algorithm. Energies.

[20] Huo Z., Zhang Y., Shu L., Gallimore M. (2019). A New Bearing Fault Diagnosis Method Based on Fine-to-Coarse Multiscale Permutation Entropy, Laplacian Score and SVM. IEEE Access.

[21] Piltan F., Prosvirin A.E., Jeong I., Im K., Kim J.-M. (2019). Rolling-Element Bearing Fault Diagnosis Using Advanced Machine Learning-Based Observer. Appl. Sci..

[22] Lu Y., Liu Y. Recognition of rotor rubbing fault types based on BP neural networks. Proceedings of the The 27th Chinese Control and Decision Conference (2015 CCDC).

[23] Luwei K.C., Yunusa-Kaltungo A., Sha’aban Y.A. (2018). Integrated Fault Detection Framework for Classifying Rotating Machine Faults Using Frequency Domain Data Fusion and Artificial Neural Networks. Machines.

[24] Gao Z., Breikin T., Wang H. (2008). Discrete-time proportional and integral observer and observer-based controller for systems with both unknown input and output disturbances. Optim. Control Appl. Methods.

[25] Ho D.W.C., Gao Z. (2004). Proportional multiple-integral observer design for descriptor systems with measurement output disturbances. IEEE Proc. Control Theory Appl..

[26] Piltan F., Kim J.-M. (2018). Bearing Fault Diagnosis by a Robust Higher-Order Super-Twisting Sliding Mode Observer. Sensors.

[27] Njima C.B., Garna T. (2020). PIO Output Fault Diagnosis by ARX-Laguerre Model Applied to 2nd Order Electrical System. IEEE Access.

[28] Piltan F., Prosvirin A.E., Sohaib M., Saldivar B., Kim J.-M. (2020). An SVM-Based Neural Adaptive Variable Structure Observer for Fault Diagnosis and Fault-Tolerant Control of a Robot Manipulator. Appl. Sci..

[29] Moeskops P., Viergever M.A., Mendrik A.M., de Vries L.S., Benders M.J.N.L., Isgum I. (2016). Automatic Segmentation of MR Brain Images with a Convolutional Neural Network. IEEE Trans. Med. Imaging.

[30] Prosvirin A., Kim J., Kim J.-M., Park J.J., Loia V., Yi G., Sung Y. (2018). Bearing Fault Diagnosis Based on Convolutional Neural Networks with Kurtogram Representation of Acoustic Emission Signals. Advances in Computer Science and Ubiquitous Computing.

[31] Liu R., Meng G., Yang B., Sun C., Chen X. (2017). Dislocated Time Series Convolutional Neural Architecture: An Intelligent Fault Diagnosis Approach for Electric Machine. IEEE Trans. Ind. Inform..

[32] Appana D.K., Prosvirin A., Kim J.-M. (2018). Reliable fault diagnosis of bearings with varying rotational speeds using envelope spectrum and convolution neural networks. Soft Comput..

[33] Wu X., Peng Z., Ren J., Cheng C., Zhang W., Wang D. (2020). Rub-Impact Fault Diagnosis of Rotating Machinery Based on 1-D Convolutional Neural Networks. IEEE Sens. J..

[34] Chen Z., Li Z. Research on fault diagnosis method of rotating machinery based on deep learning. Proceedings of the 2017 Prognostics and System Health Management Conference (PHM-Harbin).

[35] Khan S.A., Prosvirin A.E., Kim J.-M. Towards bearing health prognosis using generative adversarial networks: Modeling bearing degradation. Proceedings of the 2018 International Conference on Advancements in Computational Sciences (ICACS).

[36] Liu Q., Ma G., Cheng C. (2020). Data Fusion Generative Adversarial Network for Multi-Class Imbalanced Fault Diagnosis of Rotating Machinery. IEEE Access.

[37] Mao W., Liu Y., Ding L., Li Y. (2019). Imbalanced Fault Diagnosis of Rolling Bearing Based on Generative Adversarial Network: A Comparative Study. IEEE Access.

[38] Charte D., Charte F., García S., del Jesus M.J., Herrera F. (2018). A practical tutorial on autoencoders for nonlinear feature fusion: Taxonomy, models, software and guidelines. Inf. Fusion.

[39] Jiang G., Xie P., He H., Yan J. (2018). Wind Turbine Fault Detection Using a Denoising Autoencoder With Temporal Information. IEEE/ASME Trans. Mechatron..

[40] Principi E., Rossetti D., Squartini S., Piazza F. (2019). Unsupervised electric motor fault detection by using deep autoencoders. IEEE/CAA J. Autom. Sin..

[41] Jiang G., He H., Xie P., Tang Y. (2017). Stacked Multilevel-Denoising Autoencoders: A New Representation Learning Approach for Wind Turbine Gearbox Fault Diagnosis. IEEE Trans. Instrum. Meas..

[42] Bengio Y., Courville A., Vincent P. (2014). Representation Learning: A Review and New Perspectives. arXiv.

[43] Guyon I., Elisseeff A., Guyon I., Nikravesh M., Gunn S., Zadeh L.A. (2006). An Introduction to Feature Extraction. Feature Extraction.

[44] García S., Luengo J., Herrera F. (2015). Data Preprocessing in Data Mining.

[45] Ali M.B. (2015). Use of Dropouts and Sparsity for Regularization of Autoencoders in Deep Neural Networks. Master’s Thesis.

[46] Klambauer G., Unterthiner T., Mayr A., Hochreiter S. (2017). Self-Normalizing Neural Networks. arXiv.

[47] Glorot X., Bengio Y. Understanding the difficulty of training deep feedforward neural networks. Proceedings of the Thirteenth International Conference on Artificial Intelligence and Statistics.

[48] Tieleman T., Hinton G. (2012). Lecture 6.5-rmsprop. COURSERA Neural Netw. Mach. Learn.

[49] Piltan F., Kim J.-M. (2020). Bearing Fault Identification Using Machine Learning and Adaptive Cascade Fault Observer. Appl. Sci..

[50] Sokolova M., Lapalme G. (2009). A systematic analysis of performance measures for classification tasks. Inf. Process. Manag..

